# Recurrent Aggressive Hepatocellular Carcinoma Presenting With Chest Wall Metastasis and Portal Vein Thrombosis: A Rare Case and a Multidisciplinary Perspective

**DOI:** 10.7759/cureus.87330

**Published:** 2025-07-05

**Authors:** Nasim Salimiaghdam, Ahmed Mustafa, Irene O Pokuaa, Afshin Hamidi, Emily Chen

**Affiliations:** 1 Internal Medicine Residency Program, Capital Health Regional Medical Center, Trenton, USA; 2 Internal Medicine, Capital Health Regional Medical Center, Trenton, USA; 3 Hematology and Medical Oncology, Capital Health Regional Medical Center, Trenton, USA

**Keywords:** chest wall metastasis, hepatitis c, hepatocellular carcinoma (hcc), portal vein tumor thrombosis, recurrence

## Abstract

Hepatocellular carcinoma (HCC) is a leading cause of cancer-related mortality worldwide, often presenting with aggressive intrahepatic and extrahepatic spread. Extrahepatic metastasis occurs in a significant proportion of advanced HCC cases, most commonly affecting the lungs, regional lymph nodes, bones, and adrenal glands. However, metastasis to the chest wall is exceedingly rare and infrequently reported in the literature.

We report an unusual case of a 71-year-old male with chronic hepatitis C infection and a history of HCC resection in 2019, who presented with abdominal distension, lower extremity edema, and a rapidly enlarging chest wall mass. Imaging and biopsy confirmed metastatic HCC, showing trabecular architecture and positive immunohistochemical staining for HepPar-1 and glypican-3, with portal vein thrombosis (PVT) and chest wall involvement - a rare metastatic pattern. This case highlights HCC’s aggressive nature, the importance of early recognition of atypical metastases, and the value of multidisciplinary management - encompassing oncology, hepatology, radiology, and pathology - in improving patient outcomes.

## Introduction

Hepatocellular carcinoma (HCC) accounts for the majority of primary liver cancers and poses a significant global health challenge due to its late diagnosis, aggressive behavior, and limited treatment options that can cure the disease [1,2]. Although there have been improvements in screening programs for high-risk groups, many patients are still diagnosed at advanced stages, where curative treatments like surgery or transplantation are not possible. The high incidence of HCC is mainly linked to chronic liver diseases, with key risk factors being chronic infections with hepatitis B and C, alcoholic liver disease, and metabolic dysfunction-associated steatohepatitis (MASH) - previously referred to as nonalcoholic steatohepatitis (NASH) - all of which lead to liver inflammation and fibrosis [2-4]. The introduction of direct-acting antivirals (DAAs) has notably decreased the occurrence of hepatitis C virus (HCV)-related HCC, but a significant number of cases still develop in patients with established cirrhosis, highlighting the need for ongoing monitoring in these groups [5].

HCC is particularly known for its tendency to invade blood vessels, especially the portal venous system. Tumor-associated portal vein thrombosis (PVT) is found in up to 50% of advanced HCC cases and indicates a poor prognosis, as it severely restricts treatment options and increases the risk of liver failure and portal hypertension [6]. In HCC, extrahepatic metastases often occur via hematogenous dissemination, lymphatic invasion, and potential seeding through Batson’s plexus [7-9]. These pathways contribute to metastases commonly involving the lungs, lymph nodes, and bones. However, chest wall metastasis is exceptionally rare, with fewer than 20 cases reported in the literature [10]. The reasons behind these unusual metastatic patterns are not well understood but likely involve a combination of hematogenous spread, direct invasion, and tumor microenvironmental factors - such as vascular endothelial growth factor (VEGF) and epithelial-mesenchymal transition (EMT) pathways - that promote aggressive dissemination [11].

This report outlines a rare and aggressive form of metastatic HCC, specifically noting the involvement of the chest wall along with tumor-associated PVT. Presenting such a case offers both clinical and academic value, highlighting the importance of recognizing atypical metastatic routes and promoting a multidisciplinary approach to diagnosis and treatment.

## Case presentation

A 71-year-old male with a known history of chronic hepatitis C infection and previously treated HCC, status post-hepatic resection in 2019, presented with new symptoms. Following surgical resection of HCC in 2019, the patient underwent routine surveillance with abdominal ultrasound and serum alpha-fetoprotein (AFP) every six months, consistent with standard guidelines. Surveillance was maintained until he presented clinically with symptoms and imaging suggestive of recurrence approximately four years later. He reported progressive abdominal distension, exertional shortness of breath, and the recent development of a rapidly enlarging mass on the right side of his chest over the preceding month. He also endorsed intermittent episodes of non-bloody diarrhea but denied associated symptoms such as fever, hematemesis, or melena. The patient’s past medical history included chronic HCV infection, treated with DAAs resulting in sustained virologic response (SVR); a history of HCC treated with surgery; ventral hernia repair in 2022; and long-standing hypertension. He presented with an Eastern Cooperative Oncology Group (ECOG) performance status of 2.

On physical examination, the patient’s vital signs were stable. The abdominal assessment revealed moderate distension with shifting dullness, bilateral pitting edema, and no splenomegaly - findings consistent with portal hypertension likely due to chronic liver disease rather than metastatic spread. He had bilateral pitting edema of the lower extremities, more pronounced on the left side. A firm, non-tender mass was palpable on the right lateral chest wall, measuring approximately 4.1 × 5.5 × 5.3 cm. Histopathological examination of the biopsy confirmed a moderately differentiated HCC, without features suggestive of fibrolamellar, clear cell, or other uncommon variants.

The liver’s radiological assessment showed a nodular liver surface with coarse echotexture and segmental atrophy, consistent with cirrhosis. There was no radiological evidence of intrahepatic metastatic lesions or hepatic venous outflow obstruction. Considering portal vein patency and the absence of focal liver lesions except for the primary mass, we attribute the liver’s decompensated state to cirrhosis rather than metastasis.

Laboratory investigations showed an elevated AFP level of 68.7 nanograms per milliliter (ng/mL) (reference: <7 ng/mL) and a CA19-9 level of 231 units per milliliter (U/mL) (reference: <37 U/mL). Carcinoembryonic antigen (CEA) was within normal limits at 4.9 ng/mL. The HCV RNA level was 110,000 international units per milliliter (IU/mL). Liver function tests demonstrated mild elevations in aspartate aminotransferase (AST) and alanine aminotransferase (ALT), while bilirubin and alkaline phosphatase levels remained within normal limits. Additional liver function parameters - albumin, 3.0 grams per deciliter (g/dL); international normalized ratio (INR), 1.3; and platelet count, 102,000 per microliter (µL) - indicated impaired hepatic reserve, consistent with Child-Pugh Class B.

PIVKA-II (protein induced by vitamin K absence-II) testing was not performed during the diagnostic workup. The patient had no prior history of ablative therapies, such as radiofrequency ablation or transarterial chemoembolization (TACE), before undergoing surgical resection. He also denied any history of alcohol use, and, apart from chronic hepatitis C, he had no other significant comorbid medical conditions. At the time of presentation, the patient had a Child-Pugh score of 7 (Class B), indicating moderately impaired liver function. The Barcelona Clinic Liver Cancer (BCLC) stage was C, consistent with portal vein invasion and extrahepatic spread.

**Figure 1:**
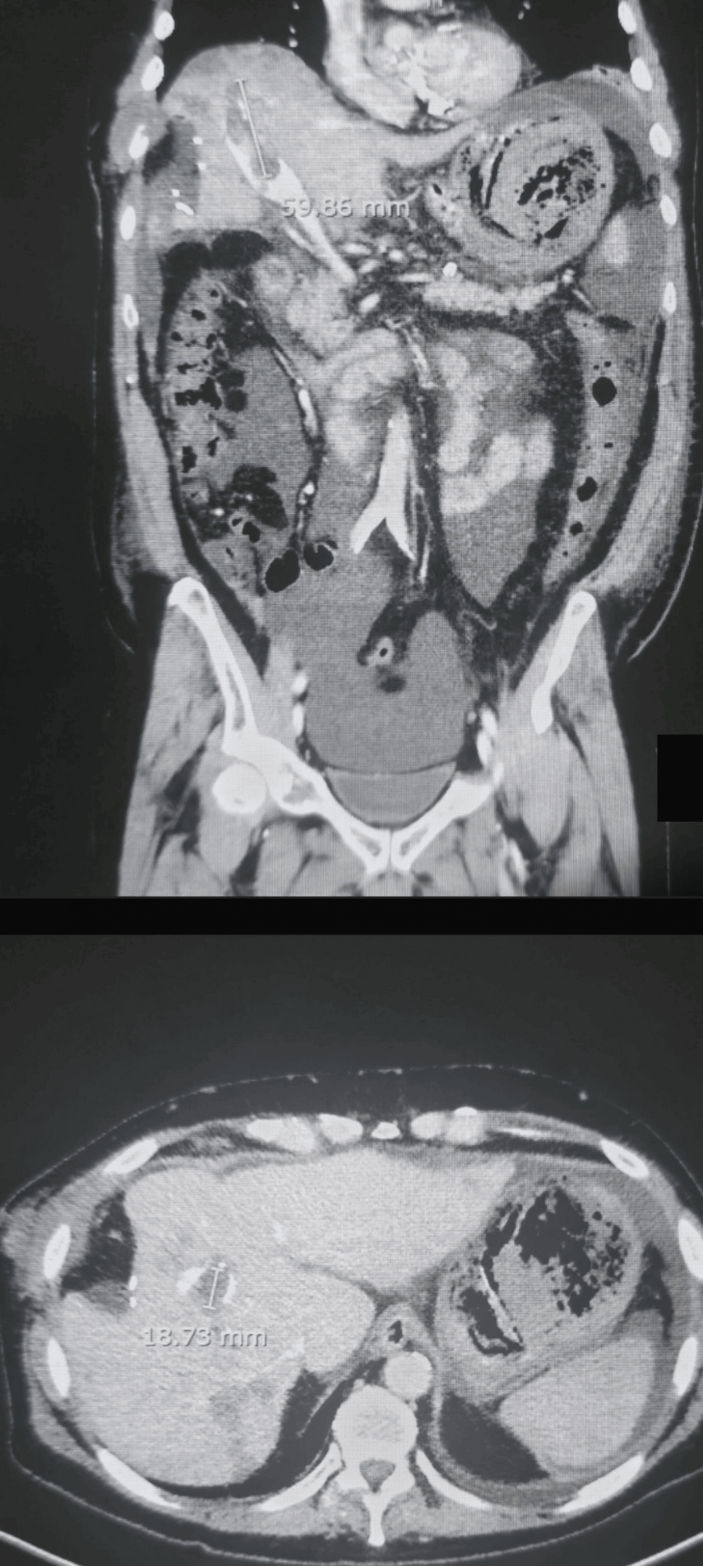
CT images showing hepatic metastasis and tumor-associated right portal vein thrombosis. CT, computed tomography

**Figure 2:**
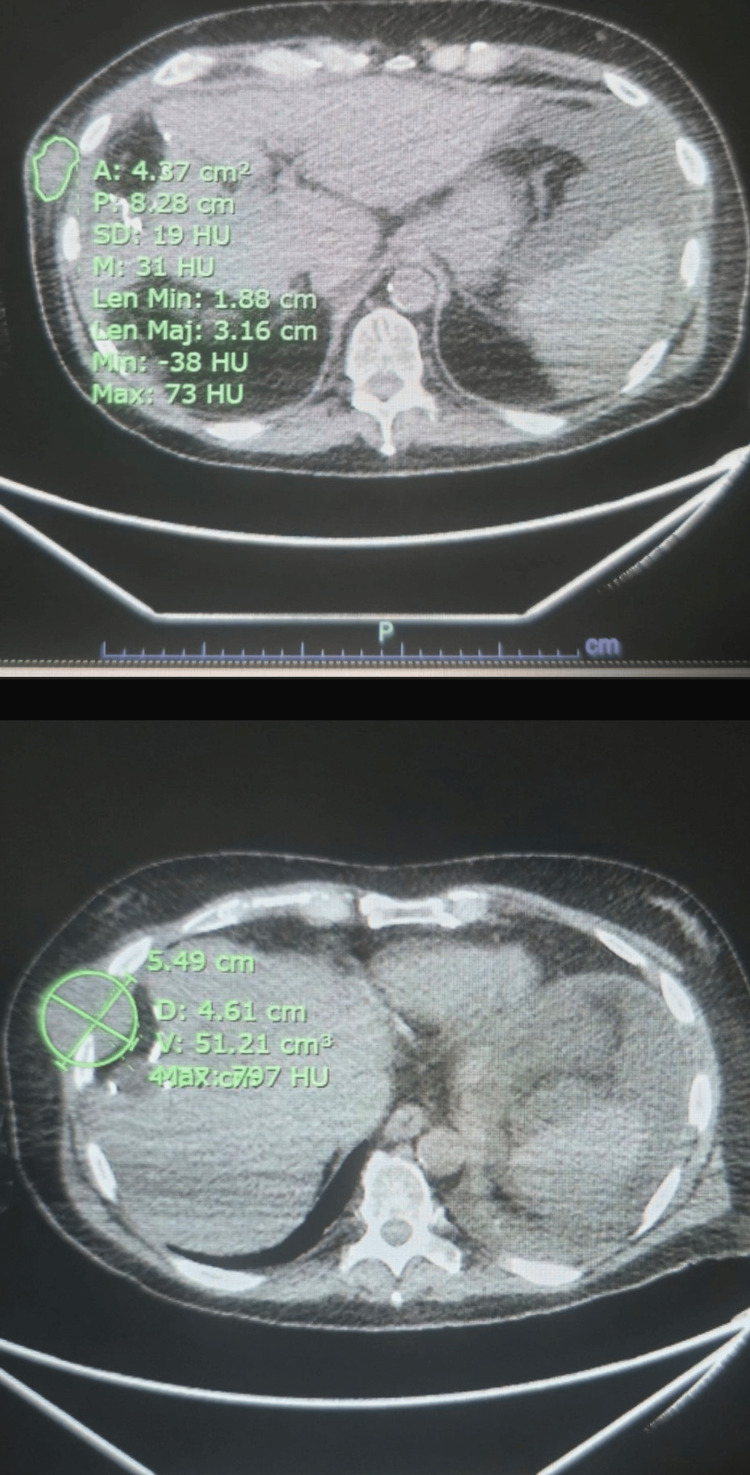
CT images demonstrating chest wall metastasis of HCC with rib involvement and left pleural effusion. CT, computed tomography; HCC, hepatocellular carcinoma

An abdominopelvic computed tomography (CT) contrast-enhanced scan revealed right PVT with arterial phase enhancement, consistent with malignant thrombus due to HCC infiltration, and a hypoattenuating lesion in segment 4A of the liver, measuring 2.3 cm (Figure 1). Additionally, moderate ascites was noted. This imaging finding, combined with concurrent metastatic lesions and the patient’s history of advanced HCC, supported the diagnosis of metastatic PVT rather than bland thrombus. A separate soft tissue mass was observed on the right lateral chest wall, involving the sixth and seventh ribs. Subtle lucency was seen in the seventh rib, raising suspicion for possible osseous involvement. The scan also showed a left-sided pleural effusion with associated compressive atelectasis (Figure 2).

Diagnostic procedures included a diagnostic paracentesis, which revealed no evidence of malignancy; cytological analysis showed benign mesothelial cells, macrophages, and lymphocytes. We conducted further diagnostic procedures, including a diagnostic paracentesis that revealed no evidence of malignancy, with cytology showing benign mesothelial cells, macrophages, and lymphocytes. The serum-ascites albumin gradient (SAAG) was calculated at 1.8 g/dL (serum albumin: 3.0 g/dL; ascitic fluid albumin: 1.2 g/dL), consistent with a diagnosis of portal hypertension-related ascites.

A core needle biopsy of the chest wall mass was performed, and histopathological examination confirmed the diagnosis of metastatic HCC, showing trabecular architecture and positive immunohistochemical staining for HepPar-1 and glypican-3. A brief differential diagnosis included soft tissue sarcoma, abscess, and primary chest wall tumors, but this was ruled out by histopathology. Table 1 highlights the important laboratory results.

**Table 1:** Summary of key laboratory values at presentation. AFP, Alpha-Fetoprotein; CA 19-9, Carbohydrate Antigen 19-9; CEA, Carcinoembryonic Antigen; HCV RNA, Hepatitis C Virus Ribonucleic Acid; AST, Aspartate Aminotransferase; ALT, Alanine Aminotransferase

Laboratory Test	Value	Reference Range	Interpretation
AFP	68.7 ng/mL	<7 ng/mL	Elevated
CA 19-9	231 U/mL	<37 U/mL	Elevated
CEA	4.9 ng/mL	<5 ng/mL	Normal
HCV RNA	110,000 IU/mL	N/A	Elevated
AST/ALT	Mild elevation	N/A	Mildly elevated
Bilirubin	Normal	N/A	Normal
Alkaline Phosphatase	Normal	N/A	Normal

## Discussion

HCC is known for being one of the most vascularized solid tumors, often showing a strong tendency for vascular invasion, especially into the portal venous system. Tumor thrombi in the portal vein can be found in up to 50% of patients with advanced HCC, which significantly worsens prognosis by facilitating intrahepatic tumor spread and increasing complications related to portal hypertension, such as ascites and variceal bleeding [6,11]. Unlike bland thrombi, which result from a hypercoagulable state, tumor thrombi arise from neoplastic invasion [5]. While anticoagulation is key for bland thrombi, tumor thrombi may require TACE or systemic therapies. The effectiveness of anticoagulation in tumor-associated PVT is still debated, due to the heightened risk of bleeding in cirrhotic patients and the unclear effects on survival outcomes [12,13].

The metastatic behavior of HCC varies, with extrahepatic spread occurring in about 30%-50% of cases at autopsy [6]. The most frequently affected areas include the lungs, lymph nodes, bones, and adrenal glands, while chest wall involvement is quite rare. The exact mechanism by which HCC spreads to the chest wall is not fully understood, but possible explanations include hematogenous dissemination through the vertebral venous plexus (Batson's plexus), direct invasion, and lymphatic spread. Chest wall metastasis may present with localized pain, mass formation, and rib destruction, as seen in our patient. Differential diagnoses include soft tissue sarcoma, requiring biopsy confirmation [10,11].

Current treatment options for advanced HCC with extrahepatic metastases have advanced rapidly following a new era of targeted therapies and immunotherapies. Recently, the combination of atezolizumab, a PD-L1 (programmed death-ligand 1) inhibitor, and bevacizumab, a VEGF inhibitor, has been shown to improve overall survival when compared to sorafenib, the previous standard-of-care first-line therapy for advanced HCC. The IMbrave150 study noted improvements in overall survival and progression-free survival versus sorafenib, establishing atezolizumab and bevacizumab as the new preferred first-line regimen in eligible patients [14]. Moreover, multi-tyrosine kinase inhibitors like lenvatinib and cabozantinib have proven effective in managing disease progression in patients with extrahepatic spread [15,16]. In situations where localized metastatic disease is identified, radiotherapy has been considered a palliative measure to decrease tumor burden and relieve symptoms [17].

The patient’s elevated CA 19-9 (carbohydrate antigen 19-9) likely reflects cholestasis or inflammation rather than an HCC marker. Given the extensive disease in our patient, we started systemic therapy with lenvatinib, selected due to contraindications to atezolizumab/bevacizumab from the risk of bleeding in the setting of PVT, and planned further imaging to evaluate disease progression and inform subsequent treatment. Radiotherapy was also considered for the palliation of the chest wall mass in our case.

The potential for localized therapies, such as TACE or stereotactic body radiotherapy (SBRT), is still being actively researched, especially in cases with oligometastatic disease [17,18]. This case emphasizes several important lessons: (1) the need to consider unusual metastatic sites in patients with recurrent HCC; (2) the prognostic relevance of tumor-associated PVT; (3) treatment selection based on liver function, performance status, and bleeding risk; and (4) the evolving treatment landscape, including IO-TKI (immuno-oncology tyrosine kinase inhibitor) combinations, SBRT, and novel biomarkers.

We present a rare case of metastatic HCC with involvement of the chest wall, likely due to the infiltrative nature of the subtype. The infiltrative subtype has certain defining characteristics that reflect its associated aggressive disease, often found at diagnosis with large vascular invasion and the highest rate of extrahepatic spread among the other subtypes [19]. In this case, we care for a solitary chest wall lesion with pathology confirmed as HCC, and emphasize the importance of recognizing metastatic disease in even rare locations in digital education. Magnetic resonance imaging (MRI) and positron emission tomography-computed tomography (PET-CT) play a key role in the diagnostic work-up. In this case, MRI is the gold standard for characterization of hepatic lesions due to its superior sensitivity, as well as its ability to determine soft tissue structures, which is useful in assessing local invasion and in delineating small satellite lesions [20].

PET-CT provides additional information in identifying hypermetabolic foci, which adds metabolic information that may uncover distant metastasis that is otherwise occult. In our patient, PET-CT helped identify the metabolically active chest wall lesion, while MRI was able to demonstrate the anatomy in detail. Using both modalities provided more confidence in the diagnosis and assisted us with guiding biopsy and treatment decisions. The markedly elevated AFP level in this case (>1000 ng/mL) also supports a diagnosis of advanced HCC. AFP elevation is commonly associated with poorly differentiated, aggressive tumors and has a positive correlation with tumor burden. At a molecular level, mechanisms suggesting that VEGF upregulation, activation of the Wnt/β-catenin pathway, or EMT contribute to a more invasive and metastatic phenotype of infiltrative HCC have all been implicated. These biological observations align and are reflected in our clinical findings and help explain the unusual metastatic pattern in this patient [21].

Future studies should aim to identify predictive markers for aggressive metastatic behavior and enhance multimodal treatment approaches to improve patient outcomes.

## Conclusions

This case highlights the aggressive vascular and metastatic behavior of HCC, including rare chest wall involvement. Recognizing these patterns early facilitates appropriate diagnostic and therapeutic planning. Incorporating hepatic reserve, imaging interpretation, histopathology, and multidisciplinary input is crucial in optimizing outcomes in advanced HCC. An extensive evaluation of hepatic reserve, followed by careful inclusion of radiologic, clinical, and pathologic findings, is still critical in setting the stage, especially in a patient without clear signs of liver dysfunction. This case highlights the importance of maintaining a high degree of clinical suspicion and conducting a comprehensive workup, including in those with no conventional markers of liver disease.
